# Experimental Investigation and Thermodynamic Calculation of Ni–Al–La Ternary System in Nickel-Rich Region: A New Intermetallic Compound Ni_2_AlLa

**DOI:** 10.3390/ma11122396

**Published:** 2018-11-28

**Authors:** Jinfa Liao, Hang Wang, Tzu-Yu Chen

**Affiliations:** 1 School of Materials Science and Engineering/Institute of Engineering Research, Jiangxi University of Science and Technology, Ganzhou 341000, China; 6720170477@mail.jxust.edu.cn; 2 Materials and Engineering Research Institute, Faculty of Science, Technology and Arts, Sheffield Hallam University, City Campus, Howard Street, Sheffield S1 1WB, UK

**Keywords:** Ni-Al-La, CALPHAD, lanthanides, thermodynamic optimization, superalloys

## Abstract

The phase equilibrium of the Ni–Al–La ternary system in a nickel-rich region was observed at 800 °C and 1000 °C using scanning electron microscopy backscattered electron imaging, energy dispersive X-ray spectrometry and X-ray diffractometry. The solubility of Al in the Ni_5_La phase was remeasured at 800 °C and 1000 °C. Herein, we report a new ternary phase, termed Ni_2_AlLa, confirmed at 800 °C. Its X-ray diffraction (XRD) pattern was indexed and space group determined using Total Pattern Solution (TOPAS), and the suitable lattice parameters were fitted using the Pawley method and selected-area electron diffraction. Ni_2_AlLa crystallizes in the trigonal system with a space group *R*3 (no. 146), a = 4.1985 Å and c = 13.6626 Å. A self-consistent set of thermodynamic parameters for the Al–La and Ni–La binary systems and the Ni–Al–La ternary system includes a Ni_2_AlLa ternary phase, which was optimized using the CALPHAD method. The calculated thermodynamic and phase-equilibria data for the binary and ternary systems are consistent with the literature and measured data.

## 1. Introduction

Nickel-based single-crystal superalloys are used extensively in the blades of power turbines in modern aero-engines because of their excellent high-temperature properties, such as high-temperature strength, excellent creep and fatigue resistance, good oxidation resistance and hot corrosion resistance [1,2,3,4]. However, as the aero-engine thrust-to-weight ratio increases, turbine engines face the challenge of higher temperatures, and a nickel-based single-crystal superalloy is required to improve their high-temperature oxidation resistance and high-temperature corrosion resistance. The excellent performance of nickel-based superalloys is attributed to the addition of elements, such as Al, Mo, Cr, W, V and Ti [1]. Among these, the addition of a small amount of the rare-earth element La can significantly improve high-temperature oxidation resistance, corrosion resistance [5,6] and manufacturing performance [7]. However, excessive addition leads to an increase in the tendency to form a topologically close-packed (TCP) phase and to deterioration in the alloy properties [8], therefore it is vital that the amount of La added is precisely controlled. Alloy design depends on the thermodynamic database [9,10] CALPHAD (CALculation of PHAse Diagram) method [11,12,13] which can reduce the experimental time, shorten the development cycle and provide theoretical guidance for material design. In this study, we consider the rare-earth element La and use the CALPHAD method to optimize the Ni–Al–La ternary system thermodynamically and to establish a nickel-based superalloy database [14] which contains the rare-earth element, La.

Limited experimental equilibrium information exists for the Ni–Al–La ternary system. In the 1980s, Abramyan [15] studied a small part of the phase-equilibrium information at 800 °C and 1000 °C in the nickel-rich corner, although no detailed experimental data were provided. Therefore, it is important and necessary to acquire phase-equilibrium information for the Ni–Al–La ternary system to obtain more accurate thermodynamic parameters and to provide effective and reasonable theoretical guidance for the design of nickel-based superalloys.

The purpose of this work was to investigate the phase equilibria relationship of the Ni–Al–La system in the nickel-rich region at 800 °C and 1000 °C by using scanning electron microscopy (SEM)-backscattered electron imaging (BSE), energy dispersive X-ray spectroscopy (EDS) and X-ray diffraction (XRD). Based on the experimental results of this study and previous literature data, a thermodynamic assessment of the Ni–Al–La system was conducted using the CALPHAD approach.

## 2. Literature Review

### 2.1. Al–La Binary System

Phase-equilibrium information on the Al–La binary system has been widely reported in the literature [16,17,18,19]. Four solid–solution phases (liquid, fcc, bcc, dhcp) and six intermetallic compounds (AlLa_3_, Al_2_La, AlLa, Al_7_La_3_, Al_3_La, Al_11_La_3_) exist, including an allotropic transformation of the Al_11_La_3_ phase. La solubility in Al solid solution was reported by Drits et al. [20]. Thermodynamic optimization of the Al–La system has been conducted by various authors [21,22,23,24,25]; however, the latest experimental data [18] were not considered in previous optimization work [21]. An associate model and modified quasichemical model were applied to the liquid phase in the work of Zhou and Napolitano [24] and Jin [25], respectively, which is not applicable in the pan-nickel database. The calculated temperature of the peritectoid reaction Al_7_La_3_ → Al_3_La + Al_2_La was 1051 °C [23], which deviates substantially from the experimental value of 1190 °C [16]. The phase diagram and thermodynamic evaluation calculated by Yin et al. [22] are consistent with the literature data, however, the Al_11_La_3_ phase is not treated as β_Al_11_La_3_ and α_Al_11_La_3_. Therefore, our work adds an allotropic transformation on this basis, and adjusts the parameters of the Al_11_La_3_ phase slightly. Furthermore, the solubility of La in the Al solid–solution is also considered.

### 2.2. Ni–La Binary System

The phase equilibria of the Ni–La binary system were measured by Zhang et al. [26], Qi et al. [27] and Buschow et al. [28]. In the assessment by Okamoto [29] and Pan et al. [30], there were four solution phases (liquid, fcc, bcc, dhcp) and nine intermediate phases (Ni_3_La_7_, NiLa_3_, NiLa, Ni_3_La_2_, Ni_16_La_7_, Ni_3_La, α-Ni_7_La_2_, β-Ni_7_La_2_, Ni_5_La). Despite a reported new Ni_19_La_5_ phase [31,32,33,34], this phase has not been taken into account in the optimization work [35,36]. Recently An et al. [37] confirmed the formation of a Ni_19_La_5_ phase from a peritectoid reaction Liq. + Ni_5_La → Ni_19_La_5_, and this has also been considered in their optimization work. Therefore, we have used most of thermodynamic parameters from reference [37] in this work. Although the mutual solubilities of Ni and La in the terminal solution phases of the La (hcp, bcc, fcc) and Ni (fcc) are very low, the high-temperature oxidation resistance of a nickel-based superalloy can be improved by La solubility. Hence, the solubility of La (0.2 ± 0.05 at.%) measured by Dischinger et al. [35] was used in this assessment.

### 2.3. Ni–Al Binary System

**Figure 1:**
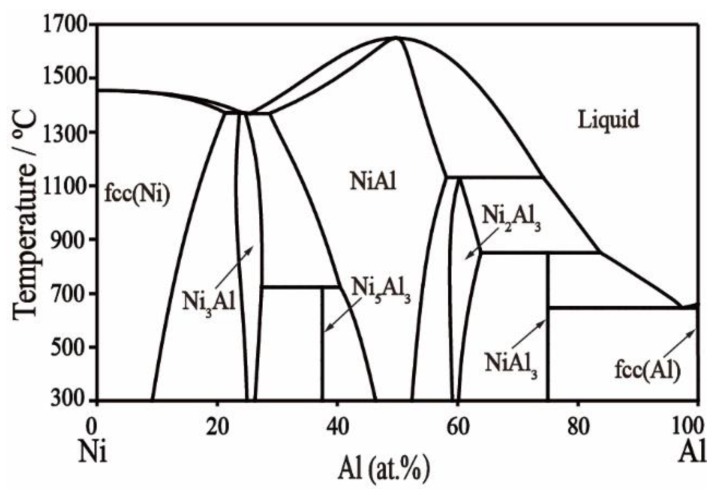
Calculated Ni–Al phase diagram.

The Ni–Al binary system has been assessed in our previous work [38] and is adjusted slightly based on Huang’s thermodynamic parameters [39]. The Ni-Al phase diagram as shown in Figure 1, and thermodynamic parameters of the Ni–Al system have been adopted from previous work without changes.

### 2.4. Ni–Al–La Ternary System

The Ni–Al–La ternary system was assessed by Ferro et al. [40] and Raghavan [41]. Two isothermal sections at the nickel-rich corner were measured at 800 °C and 1000 °C by a hardness and X-ray diffraction method, however, detailed experimental data were lacking, and only phase-equilibrium information was obtained [15]. The author also reported that the solubility of Al in the Ni_5_La phase increased with temperature and exceeded 20 at.%. Based on his paper, we found that the solubility of Al in Ni_5_La is of the order of 23 at.% at 800 °C, and increases to 25 at.% at 1000 °C. A vertical section Al_90_La—Al_90_Ni was determined by Gödecke et al. using a metallographic observation and a DTA method [42], and a ternary eutectic reaction Liq → Fcc + Al_11_La_3_ + Al_3_Ni at 625 °C was determined. Cordier et al. [43] and Takeshita et al. [44] reported the intermetallic compounds NiAlLa and Ni_4_AlLa in the nickel-rich corner. The open-source first-principle-database Open Quantum Materials Database (OQMD) [45], Materials Project [46] and Aflow (Automatic flow) [47] provide the ternary intermetallic-compound and crystal-structure information using first-principles calculation. All ternary intermetallic compounds and their crystal structures in the nickel-rich region of the Ni–Al–La ternary system from the literature and the open-source first-principle databases are listed in Table 1.

The enthalpies of mixing of the ternary liquid at different sections were measured by Feufel et al. [48] with an adiabatic calorimeter. Sommer and Schmid [49,50] adopted the same method to study the heat of the liquid Ni–Al–La alloy, but the liquidus temperature values of the Ni–Al–La alloys obtained from *Cp*–measurements were very low in comparison to the melting temperatures of the alloy, which resulted in the *Cp* values of their work exceeding the actual value. Thus, their thermodynamic optimization procedure is not utilized in this work. Additionally, Pasturl et al. [51] and Borzone et al. [52] used isoperibol calorimetry and a high-temperature direct-reaction drop calorimeter, respectively, to determine the formation of Ni_4_AlLa and NiAlLa ternary intermetallic compounds.

## 3. Materials and Methods

Four representative alloys, each weighing 15 g on an electronic balance with an accuracy of 0.0001 g, were prepared using high-purity nickel (99.99 wt.%), aluminum (99.999 wt.%) and lanthanum (99.9 wt.%) as starting materials. Mixed metals were transferred into a water-cooled copper-crucible arc furnace, vacuumed to 10^−3^ Pa and smelted in a high-purity argon (99.999%) atmosphere. Each sample was turned over and re-melted at least six times during the smelting process to ensure a homogeneous composition. The mass loss during arc smelting was less than 0.5 wt.%. The smelted sample was cut into two parts by a wire-cutting machine and sealed in quartz tubes which were evacuated and filled with high-purity argon gas for annealing at 800 °C and 1000 °C for 30 days and 15 days, respectively. Finally, the quartz tubes were quenched in cold water and broken.

The X-ray diffraction data for all alloys were obtained at room temperature using a Bruker SMART APEX II single-crystal X-ray diffractometer (Karlsruhe, Germany) with Cu *Kα*-radiation in the 2θ scan range of 10–90°. The alloy phase distributions were observed by BSE imaging of the FEI MLA650F field-emission SEM (Hillsboro, OR, USA) after standard metallographic preparation, and the elemental composition of the phases was measured by equipped EDS. The crystallographic information of the intermetallic compound was indexed using Total Pattern Solution (TOPAS) [53] software (TOPAS-Academic 6) and Pawley fits were carried out to refine lattice parameters and confirm space groups of the phases recorded before structure solution was attempted [54]. To further support the structural information acquired from XRD, we ground the alloy to below 100-μm thick and performed ion thinning (Gatan 691) and a FEI Tecnai G2 F20 (Hillsboro, OR, USA) for selected area electron diffraction (SAED).

## 4. Calculations

Pandat software [55] was used for thermodynamic calculations. The thermodynamic model for the Gibbs free energy *G* of the solution phases, including liquid, fcc, bcc and dhcp, is:

(1) Gmφ=∑i=Al,Ni,La(xiG 0iφ)+RT∑i=Al,Ni,La(xiln(xi))+G exmφ

where *i* represents elements Al, La and Ni; *x* is the composition; G 0iφ is the Gibbs free energy for pure element *i* in the structure φ phase and is obtained from Dinsdale [56]; R is the gas constant; T is the temperature in K; and G exmφ is the excess Gibbs free energy, which is described as:

(2) G exmφ=xAlxNiLAl,Niφ+xAlxLaLAl,Laφ+xLa,xNiLLa,Ni φ+xAlxNixLaLAl,Ni,Laφ

where $L_{\mathrm{Al},\mathrm{Ni}}^{\varphi }$, $L_{\mathrm{Al},\mathrm{La}}^{\varphi }$ and $L_{\mathrm{La},\mathrm{Ni}\;}^{\varphi }$ are the interaction parameters in the Al–Ni, Al–La and La–Ni binary systems and the $L_{\mathrm{Al},\mathrm{Ni},\mathrm{La}}^{\varphi }$ corresponds to the interaction parameters of the Ni–Al–La ternary system. All parameters were obtained by optimizing the experimental data.

Thermodynamic models for most of the binary phases were taken from previous work [22,37,38] except for the Ni_5_La phase. Because of the solubility of Al in the Ni_5_La phase, the thermodynamic model for the Ni_5_La phase was modified to:

(3) $$G^{{\left(\mathrm{Al},\mathrm{Ni}\right)}_{5}\mathrm{La}}=y_{\mathrm{Al}}G_{\mathrm{Al}:\mathrm{La}}+y_{\mathrm{Ni}}G_{\mathrm{La}:\mathrm{Ni}}+5RT\left(y_{\mathrm{Al}}\ln\left(y_{\mathrm{Al}}\right)+y_{\mathrm{Ni}}\ln\left(y_{\mathrm{Ni}}\right)\right)+y_{\mathrm{Al}}y_{\mathrm{Ni}}L_{\mathrm{La}:\mathrm{Al},\mathrm{Ni}}$$

where $y_{\mathrm{Al}}$ and $y_{\mathrm{Ni}}$ are the site fractions of Al and Ni in the first sublattice; $G_{\mathrm{Al}:\mathrm{La}}$ and $G_{\mathrm{La}:\mathrm{Ni}}$ are Gibbs free energies for end-members and $L_{\mathrm{La}:\mathrm{Al},\mathrm{Ni}}$ is the interaction term between the two sublattices to be optimized.

Ternary intermetallic Ni_m_Al_n_La_l_ phases were treated as stoichiometric compounds, and the Gibbs free energy was expressed as:

(4) GAl:Ni:LaNi2AlLa =nxAlG  Alfcc+l  xLaGLadhcp+m   xNi GNifcc+A+BT

where *A* and *B* are the thermodynamic parameters to be optimized.

## 5. Results and Discussion

### 5.1. Microstructure and Phase Equilibria

**Figure 2:**
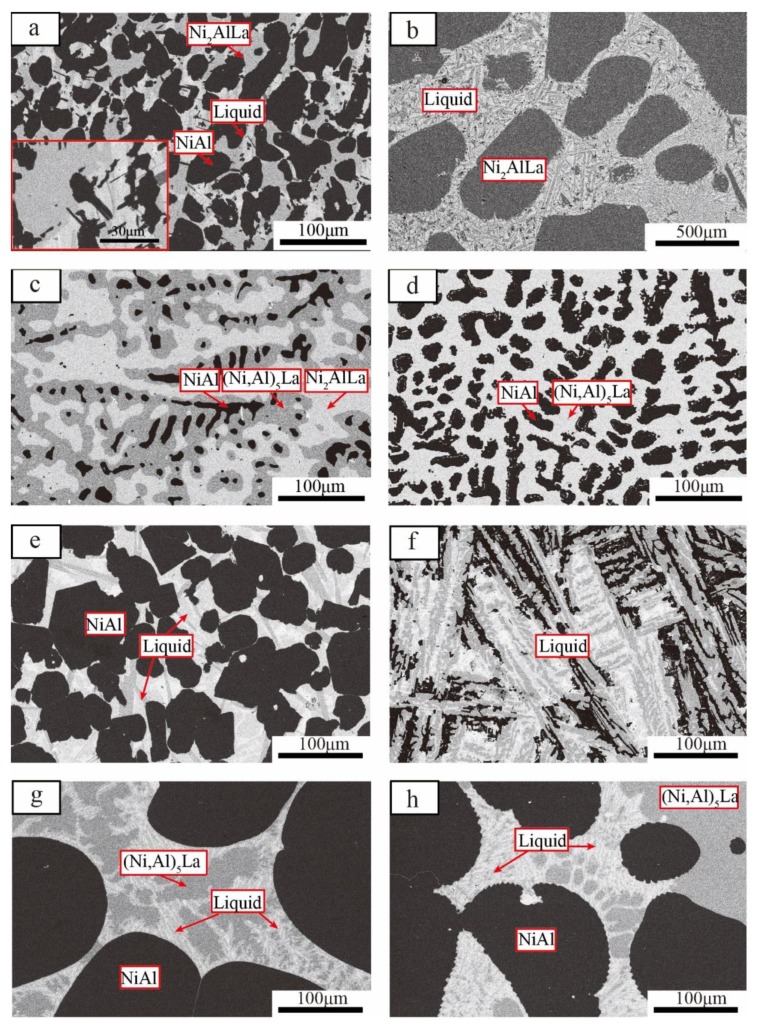
BSE images of ternary alloys annealed at 800 °C for 30 days: (**a**) #1 alloy, (**b**) #2 alloy, (**c**) #3 alloy, (**d**) #4 alloy. BSE images of ternary alloys annealed at 1000 °C for 15 days: (**e**) #1 alloy, (**f**) #2 alloy, (**g**) #3 alloy, (**h**) #4 alloy.

BSE images of the Ni–Al–La ternary alloys are shown in Figure 2, and EDS results in Table 2. All components were expressed in atomic percentage. #1 alloy exhibits a three-phase equilibrium at 800 °C as shown in Figure 2a. The dark area indicates a NiAl phase and the light-grey area is an unknown phase. A few bright-phase regions contain very fine precipitates and irregular stripes, which are likely to be the liquid phase prior to alloy removal from the furnace, and fine precipitates and irregular stripes formed during solidification. The unknown phase is a new phase consisting of 52.63Ni–24.02Al–23.36La based on the EDS result. This phase indicates a Ni_2_AlLa ternary phase that has not been measured experimentally, but only calculated using an ab initio method in the Ni–Al–La ternary system of the OQMD and Aflow database. Figure 2b shows the two-phase microstructure (Ni_2_AlLa + liquid) of the #2 alloy annealed at 800 °C for 30 days. The light-grey region is an unknown phase and is likely the Ni_2_AlLa phase from the EDS result (53.47Ni–23.74Al–22.79). The remaining stripes are precipitated by liquid during cooling. Figure 2c shows the three-phase region of #3 alloy at 800 °C. The dark area indicates a NiAl phase, where the dark-grey area is the (Ni, Al)_5_La phase and the light-grey area is an unknown phase. This light-grey component is likely to be 51.83Ni–24.30Al–23.86La from the EDS result, and it is suggested that it is the same phase, Ni_2_AlLa, observed in the #1 and #2 alloys. Figure 2d presents the two-phase equilibrium microstructure in the #4 alloy annealed at 800 °C for 30 days. The dark area is the NiAl phase, and the dark-grey area is the (Ni,Al)_5_La phase. Figure 2e shows the presence of a two-phase region (NiAl + liquid) for the #1 alloy annealed at 1000 °C for 15 days. The dark region is the NiAl phase and an irregular stripe is formed during the cooling of the liquid phase. Figure 2f shows the BSE image of the #2 alloy annealed at 1000 °C for 15 days. All stripes were formed during liquid solidification, so this is a single-phase region. Figure 2g,h represent the BSE images of #3 and #4 alloys, respectively, annealed at 1000 °C for 15 days, which represent the three-phase region (NiAl + (Ni,Al)_5_La + liquid). The dark region is the NiAl phase, the dark-grey area is the (Ni,Al)_5_La phase, the bright region contains very fine precipitates, and the irregular stripes were the liquid phase prior to the alloy removal from the furnace.

(Ni,Al)_5_La is a solid solution, based on the Ni_5_La binary phase. The (Ni,Al)_5_La phase exists in the #3 and #4 alloys. According to the corresponding EDS results, the maximum solubility of Al in Ni_5_La is 23.53 at.% at 800 °C, which is consistent with the experimental value (23.53 at.%) reported by Abramyan in 1979 [15]. When the temperature reaches 1000 °C, the maximum solubility of Al in Ni_5_La is in the order of 19.78 at.%, which is much less than the 25 at.% reported by Abramyan. Because of the unreliable experimentation and the lack of detailed experimental data in Abramyan’s results [15], the value of solid solution of Al in Ni_5_La is not convincing. Thus, the maximum solubility of Al in Ni_5_La from our present work is more reliable as the key experiment was done. The reason the solubility of Al in Ni_5_La at 1000 °C is less than that at 800 °C is mainly because with a temperature increase to 1000 °C, the liquid is more stable, which reduces the solubility of Al in the Ni_5_La phase.

### 5.2. Phase Determination

**Figure 3:**
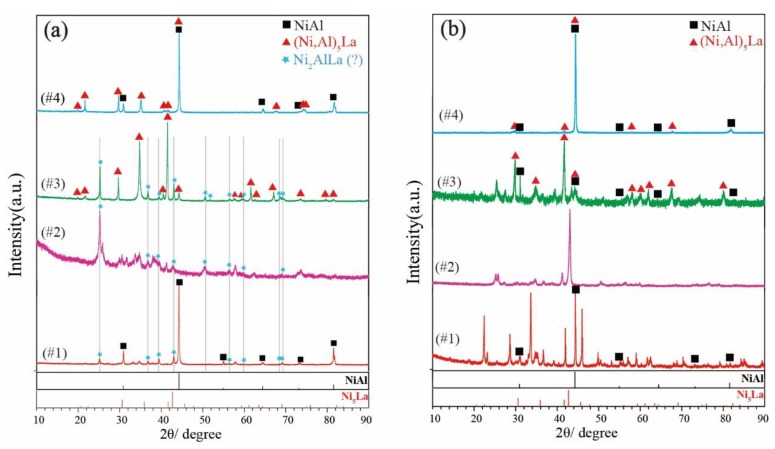
XRD patterns from: (**a**) Ni_50_Al_35_La_15_ (#1), Ni_50_Al_18_La_32_ (#2), Ni_57_Al_23_La_20_ (#3) and Ni_60_Al_30_La_10_ (#4) alloy annealed at 800 °C for 30 days; and (**b**) Ni_50_Al_35_La_15_ (#1), Ni_50_Al_18_La_32_ (#2), Ni_57_Al_23_La_20_ (#3) and Ni_60_Al_30_La_10_ (#4) alloy annealed at 1000 °C for 15 days.

Figure 3 shows the XRD patterns obtained from #1 to #4 alloys annealed at 800 °C for 30 days and 1000 °C for 15 days. The #1 alloy exists in the three-phase region (NiAl + Ni_2_AlLa + liquid). A NiAl phase was identified, and the remaining peaks result from the Ni_2_AlLa phase and the liquid phase during cooling. The #2 alloy exists in the two-phase region (Ni_2_AlLa + liquid) at 800 °C. Its corresponding XRD pattern is shown in Figure 3a. All peaks result from the Ni_2_AlLa phase and the liquid phase during cooling. The three-phase region, NiAl + (Ni,Al)_5_La + Ni_2_AlLa, exists in the #3 alloy that was annealed at 800 °C. It contains two phases in the XRD pattern Figure 3a, (Ni,Al)_5_La phase labelled with a triangle and possibly a new Ni_2_AlLa phase. The NiAl phase was not observed due to the small amount. The potential characteristic peaks of the Ni_2_AlLa phase in the #3 alloy annealed at 800 °C (marked with stars) were also found in the XRD pattern of the #1 and #2 alloys annealed at 800 °C. The #4 alloy is a two-phase region, and consists of the NiAl + (Ni,Al)_5_La phase; all phases were confirmed. All alloy phases in the #1 to #4 alloys that were annealed at 1000 °C for 15 days have been identified as shown in Figure 3b, except the liquid phase. The unknown remaining peaks result from the liquid phase, because liquid-phase solidification is a complex phase-transition process.

A new phase, termed Ni_2_AlLa, with a stoichiometric ratio of 2:1:1 (Ni:Al:La) was determined for the first time. It was observed in SEM-BSE, as shown in Figure 2 in the #1, #2 and #3 alloys annealed at 800 °C, as well as in the XRD presented in Figure 3a. Potential characteristic peaks of the Ni_2_AlLa phase are indicated with stars. The crystal structure of the Ni_2_AlLa phase was calculated by the ab intio method in the Ni–Al–La ternary system of the OQMD and Aflow database, and the calculated structures are listed in Table 1. The XRD pattern of the Ni_2_AlLa phase calculated by the OQMD and Aflow database match our experimental measurements, so the crystal structure solution of the Ni_2_AlLa phase was attempted. TOPAS was used to index the potential characteristic peaks of the Ni_2_AlLa phase for possible lattice constants and space group. The suitable cell parameters and space groups were fitted using the whole powder pattern decomposition (WPPD) Pawley method. After satisfying Pawley fits of various candidates were achieved, we narrowed down to two different crystal structures for the new Ni_2_AlLa intermetallic compound. Selective candidate cells are listed in Table 3. To confirm the crystal information from the XRD pattern is challenging at this stage due to the poor quality of the experimental XRD pattern. Furthermore, a great peak selection, which is essential for indexing, is hard to perform due to the peaks overlapping from multiple-phase components. To further confirm the crystal information of this phase, SAED using TEM of the #3 alloy annealed at 800 °C was carried out.

**Figure 4:**
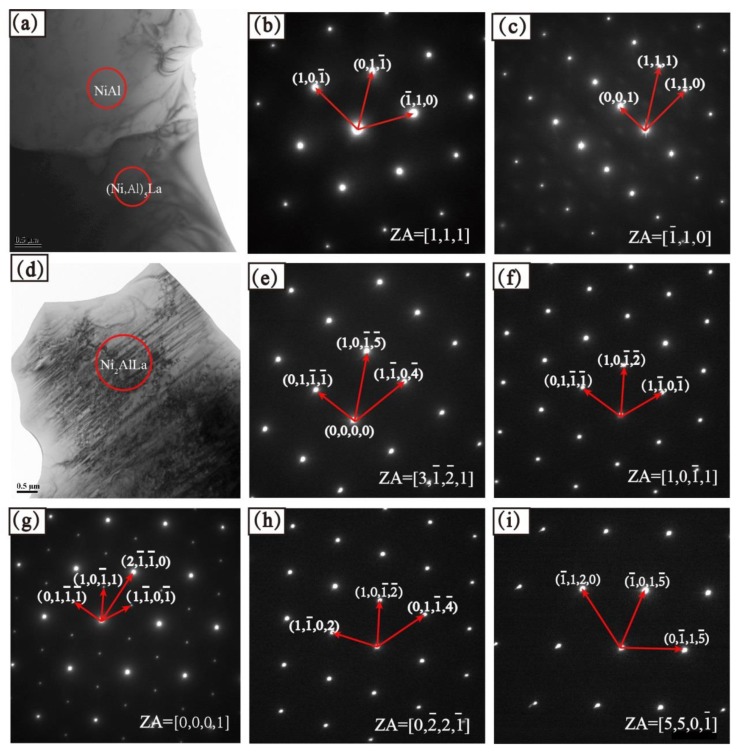
Crystal morphology and SAED patterns of #3 alloy annealed at 800 °C for 30 days. (**a**) crystal morphology of NiAl and (Ni, Al)_5_La phase, (**b**) SAED image of NiAl phase with zone axis of [111], (**c**) SAED image of (Ni,Al)_5_La phase with zone axis of [$\bar{1}$11], (**d**) crystal morphology of Ni_2_AlLa phase, (**e**) SAED image of Ni_2_AlLa phase with zone axis of [3$\bar{1}$$\bar{2}$1], (**f**) SAED image of Ni_2_AlLa phase with zone axis of [10$\bar{1}$1], (**g**) SAED image of Ni_2_AlLa phase with zone axis of [0001], (**h**) SAED image of Ni_2_AlLa phase with zone axis of [0$\bar{2}$2$\bar{1}$], (**i**) SAED image of Ni_2_AlLa phase with zone axis of [550$\bar{1}$].

**Figure 5:**
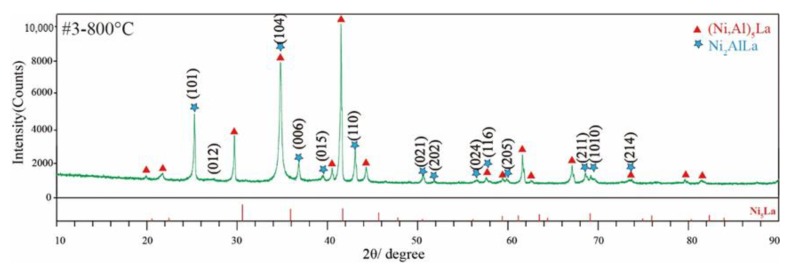
XRD pattern of #3 alloy annealed at 800 °C for 30 days.

Figure 4 presents the crystal morphology and SAED patterns of the #3 alloy annealed at 800 °C for 30 days, and indicates that a three-phase region, NiAl + (Ni,Al)_5_La and Ni_2_AlLa phase, existed. The calibration results of the SAED of the NiAl and Ni_5_La phase are shown in Figure 4b,c. Figure 4b shows the diffraction spot of the NiAl phase on the [111] zone axis, and the diffraction spot of the (Ni,Al)_5_La phase on the [$\bar{1}$11] zone axis is exhibited in Figure 4c. For the Ni_2_AlLa new phase, the SAED images of five different crystal zone axes were taken as shown in Figure 4e,f. The orthorhombic and hexagonal/trigonal cell choices obtained using TOPAS (Table 3) were used to calibrate the SAED image of the five different zone axes. It is impossible to calibrate any one of the SAED images using any of the two sets of lattice parameters of the orthorhombic system. Furthermore, the crystal structure of the Ni_2_AlLa ternary phase calculated by the OQMD and Aflow database cannot calibrate any of the electron diffraction images. All electron-diffraction images were indexed and showed high agreement with the cell in the trigonal system *R*3 space group, with the indexing result presented in Figure 4e,f. Based on the EDS, XRD and SAED results, a new Ni_2_AlLa phase has been confirmed. The crystal structure of the Ni_2_AlLa intermetallic compound was trigonal, with a space group *R*3 (no. 146) and a = 4.1985 Å, c = 13.6626 Å. The assignment of each reflection of the Ni_2_AlLa phase with indices *hkl* based on the indexing result is shown in Figure 5.

### 5.3. Thermodynamic Calculation

**Figure 6:**
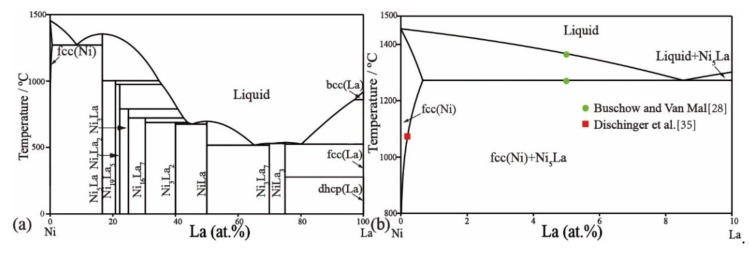
Calculated Ni–La phase diagram. (**a**) Full composition range, (**b**) magnified Ni-rich corner showing solubility of La in fcc(Ni).

**Figure 7:**
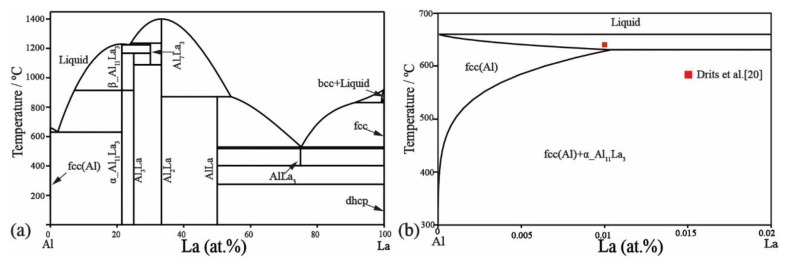
Calculated Al–La phase diagram. (**a**) Full composition range, (**b**) magnified Ni-rich corner showing solubility of La in fcc(Al).

Table 4 lists the optimized thermodynamic parameters for the Ni–Al binary system using the phase diagram as calculated in Figure 1. The phase diagram was calculated for the Ni–La binary system and is shown in Figure 6a. The solubility of La in the fcc Ni solution has been added, which fits the literature data [35] as shown in Figure 6b. The invariant reactions have not been affected, except the eutectic reaction Liq → fcc-Ni + Ni_7_La_2_. The temperature (1272 °C) of this eutectic reaction increases by less than 4 °C compared with the reference [41] and is closer to the experimental data (1275 °C) [28]. The calculated phase diagram of the Al–La binary system is shown in Figure 7a. The solubility of La in the fcc-Al phase is also considered to be ~0.01 at.%, which is consistent with the experimental data [20] in Figure 7b. The Al_11_La_3_ phase is divided into two phases, α_Al_11_La_3_ and β_Al_11_La_3_, according to their crystal structure stable at low and high temperature, respectively. The transformation temperature between the two Al_11_La_3_ phases is 915 °C, which is the same as that reported in the literature [16]. The temperature of the eutectic reaction Liq → Fcc + α-Al_11_La_3_ is calculated to be 631 °C, which is consistent with the 634 °C reported by Kononenko and Golubev [18]. Other invariant reactions remain unchanged. Overall, the calculated phase diagram in this work reproduces the literature data well.

**Figure 8:**
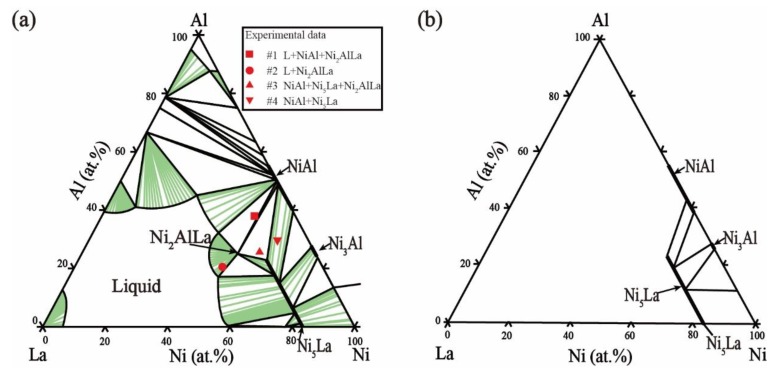
Isothermal section of the Ni–Al–La ternary system at 800 °C. (**a**) Our calculated isothermal section compared with our experimental results, (**b**) literature data [15].

**Figure 9:**
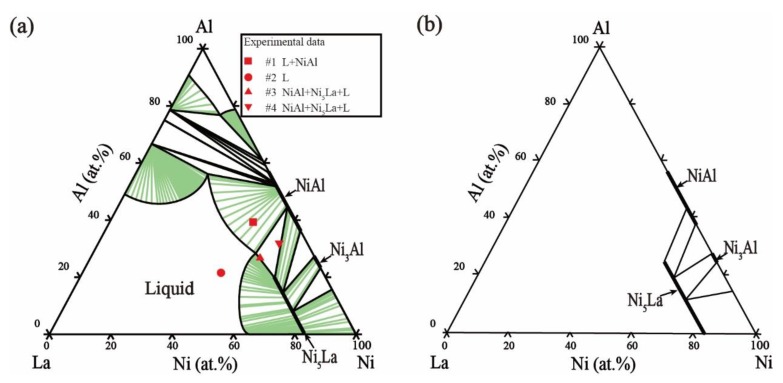
Isothermal section of the Ni–Al–La ternary system at 1000 °C. (**a**) Our calculated isothermal section compared with the experimental results, (**b**) literature data [15].

**Figure 10:**
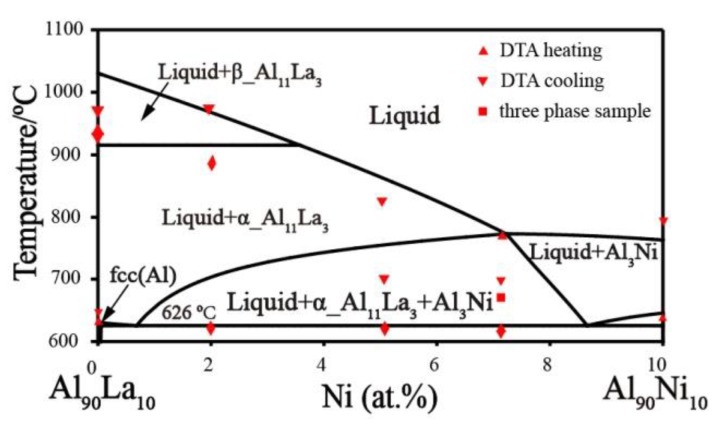
Calculated vertical section of Al_90_La–Al_90_Ni compared with the experimental data [42].

**Figure 11:**
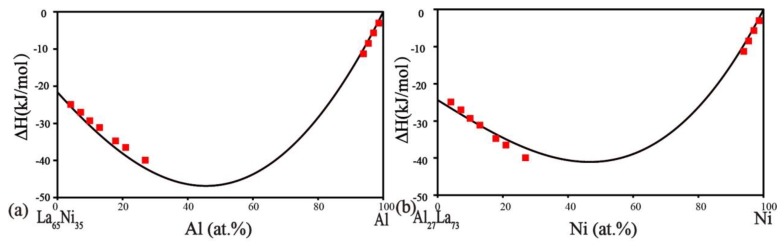
Calculated mixing enthalpies of the Ni–Al–La ternary system at 800 °C compared with experimental data [49], (**a**) (La_65_Ni_35_)_1−x_Al_x_ section; (**b**) (Al_27_La_73_)_1−x_Ni_x_ section.

The thermodynamic parameters of the Ni–Al–La ternary system optimized based on our experimental data and experimental information from the literature, are listed in Table 4. The calculated isothermal sections at 800 °C and 1000 °C are shown in Figure 8a and Figure 9a, where the red marks indicate the current experimental data. The phase-equilibrium information calculated for the isothermal sections shows high agreement with current experimental data. The calculated solubility of Al in the Ni_5_La phase is 22.82 at.% and 19.72 at.% at 800 °C and 1000 °C, respectively, which is consistent with our current EDS results. The calculated phase equilibrium of the vertical section at Al_90_La–Al_90_Ni, as shown in Figure 10, matches the experimental literature data [42]. The eutectic reaction Liq → Fcc + Al_11_La_3_ + Al_3_Ni temperature calculated in our work is 1 °C higher than the experimental value of 625 °C [42]. In general, the calculated phase-equilibrium information is consistent with both the previous literature and current experimental information. The calculated liquid-phase mixing of the different cross sections of the Ni–Al–La ternary is shown in Figure 11 compared with Reference [49], and it is clear that our results match those in the literature.

## 6. Conclusions

1. The nickel-rich-region isothermal sections of the Ni–Al–La ternary system were updated at 800 °C and 1000 °C. The maximum solubility of Al in Ni_5_La was in the order of 23.53 at.% at 800 °C. When the temperature increased to 1000 °C, the maximum solubility of Al in Ni_5_La was 19.84 at.%.
2. A new phase, termed Ni_2_AlLa, has been discovered experimentally and confirmed for the first time. The structural information of the new ternary intermetallic compound Ni_2_AlLa was determined. The investigated compound crystallizes in the trigonal system, space group *R*3 (no. 146) with a = 4.1985 Å, c = 13.6626 Å.
3. Based on the current experimental data and experimental information reported in the literature, a thermodynamic optimization of the Ni–Al–La ternary system was carried out using the CALPHAD method. The solubility of La in the fcc (Ni) and fcc (Al) and an allotropic transformation of the Al_11_La_3_ phase were considered, and the Al–La and Ni–La binary systems were re-optimized. All optimized results and experimental information reflect good consistency. This work can be used as part of a thermodynamic database of multicomponent nickel-based alloys.

## Figures and Tables

**Table 1:** Crystal structure of ternary compound in nickel-rich corner of Ni–Al–La ternary system from different sources.

Crystal System	Space Group	Structure Type	Lattice Constants (Å)			Source
			a	b	c	
Ni_9_Al_2_La	*Cmmm*	-	8.599	5.040	8.062	OQMD
	*F*1	-	5.048	8.599	8.035	Materials project
Ni_4_AlLa	*P*6*/mmm*	CaCu_5_	5.069	5.069	4.074	[51]
NiAlLa	*Pnma*	-	7.199	4.203	16.085	[52]
Ni_2_AlLa	*Fm-*3*m*	Cu_2_MnAl	6.724	6.724	6.724	OQMD
	*P*4*/mmm*	-	4.640	4.640	3.1615	Aflow
	*P*4*/mmm*	-	3.117	3.117	6.793	Aflow
	*P*4*/mmm*	-	3.074	3.074	6.900	Aflow
	*P*4*/mmm*	-	3.126	3.126	6.919	Aflow
	*Cm*	-	4.001	7.189	5.641	Aflow
	*Fm-*3*m*	-	6.536	6.536	6.536	Aflow
	*Pmm*2	-	3.129	3.159	6.875	Aflow
	*F-*43*m*	-	6.591	6.591	6.591	Aflow
	*Cmmm*	-	6.574	6.596	3.141	Aflow
	*I-*4*m*2	-	4.012	4.012	8.892	Aflow

**Table 2:** Heat-treatment conditions and equilibrium composition of the determined Ni–Al–La ternary system.

Temperature	Alloys (at.%)	Annealed Time	Phase by XRD	Phase	Composition (at.%)		
				by SEM and EDS	Ni	Al	La
800 °C	Ni_50_Al_35_La_15_ (#1)	30 days	NiAl + Ni_2_AlLa	NiAl	49.68	50.28	0.05
				Ni_2_AlLa	53.63	23.02	23.36
				Liquid	22.87	43.30	33.83
	Ni_50_Al_18_La_32_ (#2)	30 days	Ni_2_AlLa	Ni_2_AlLa	53.47	23.74	22.79
				Liquid	50.04	6.61	43.38
	Ni_57_Al_23_La_20_ (#3)	30 days	Ni_2_AlLa + Ni_5_La	NiAl	50.48	49.05	0.47
				Ni_2_AlLa	51.83	24.30	23.86
				Ni_5_La	60.77	23.53	15.37
	Ni_60_Al_30_La_10_ (#4)	30 days	NiAl + Ni_5_La	NiAl	52.33	47.50	0.17
				Ni_5_La	63.35	21.71	14.94
1000 °C	Ni_50_Al_35_La_15_ (#1)	15 days	NiAl	NiAl	51.35	48.65	0.00
				Liquid	51.05	20.38	28.57
	Ni_50_Al_18_La_32_ (#2)	15 days		Liquid	57.03	10.72	27.85
	Ni_57_Al_23_La_20_ (#3)	15 days	NiAl + Ni_5_La	NiAl	47.18	52.66	0.15
				Ni_5_La	65.09	19.78	15.13
				Liquid	55.29	17.89	26.82
	Ni_57_Al_23_La_20_ (#4)	15 days	NiAl + Ni_5_La	NiAl	46.74	53.26	0.00
				Ni_5_La	66.95	17.95	15.09
				Liquid	55.29	17.75	26.96

**Table 3:** Possible crystal structure of the Ni_2_AlLa intermetallic compound.

Crystal System	Space Group	Lattice Constants (Å)		
		a	b	c
Hexagonal/Trigonal	*P*3*c*1	10.740	10.740	3.367
	*R*3	4.189	4.189	14.663
	*P*31*c*	8.138	8.138	3.396
Orthorhombic	*F*222	9.739	10.130	2.195
	*C*222	3.263	2.733	7.021

**Table 4:** Optimized thermodynamic parameters of the Ni–Al–La ternary system.

Phase	Models	Parameters	Source
Liquid	(Al,Ni,La)	${\;}^{0}L_{\mathrm{Al},\mathrm{Ni},\mathrm{La}}^{\mathrm{Liq}}=51,547-20T$	This work
		${\;}^{1}L_{\mathrm{Al},\mathrm{Ni},\mathrm{La}}^{\mathrm{Liq}}=76,000-90\;T$	This work
		${\;}^{2}L_{\mathrm{Al},\mathrm{Ni},\mathrm{La}}^{\mathrm{Liq}}=0$	This work
fcc	(Al,Ni,La)	${\;}^{0}L_{\mathrm{Al},\mathrm{La}}^{\mathrm{Fcc}}=-63,500-10\;T$	This work
		${\;}^{1}L_{\mathrm{Al},\mathrm{La}}^{\mathrm{Fcc}}=-80,000$	This work
		${\;}^{0}L_{\mathrm{Ni},\mathrm{La}}^{\mathrm{Fcc}}=-2000$	This work
		${\;}^{1}L_{\mathrm{Ni},\mathrm{La}}^{\mathrm{Fcc}}=-58,800$	This work
bcc	(Al,Ni,La)	${\;}^{0}L_{\mathrm{Al},\mathrm{La}}^{\mathrm{Bcc}}=-68,800$	This work
α_Al_11_La_3_	(Al)_11_(La)_3_	${\;}^{0}G_{\mathrm{Al}:\mathrm{La}}^{\alpha \_{\mathrm{Al}}_{11}{\mathrm{La}}_{3}}=-740,070.1+83.031\;T+3{\;}^{0}G_{\mathrm{La}}^{\mathrm{dhcp}}+11{\;}^{0}G_{\mathrm{Al}}^{\mathrm{fcc}}$	This work
β_Al_11_La_3_	(Al)_11_(La)_3_	${\;}^{0}G_{\mathrm{Al}:\mathrm{La}}^{\beta \_{\mathrm{Al}}_{11}{\mathrm{La}}_{3}}=-738,882.1+82.031\;T+3{\;}^{0}G_{\mathrm{La}}^{\mathrm{dhcp}}+11{\;}^{0}G_{\mathrm{Al}}^{\mathrm{fcc}}$	This work
Ni_5_La	(La) (Al,Ni)_5_	${\;}^{0}G_{\mathrm{La}:\mathrm{Ni}}^{{\mathrm{Ni}}_{5}\mathrm{La}}=-168,451.2983+29.256\;T$	Ref [38]
		${\;}^{0}G_{\mathrm{La}:\mathrm{Al}}^{{\mathrm{Ni}}_{5}\mathrm{La}}=-8550+50\;T+{\;}^{0}G_{\mathrm{La}}^{\mathrm{dhcp}}+\;5^{0}G_{\mathrm{Al}}^{\mathrm{fcc}}$	This work
		${\;}^{0}L_{\mathrm{La}:\mathrm{Al},\mathrm{Ni}}^{{\mathrm{Ni}}_{5}\mathrm{La}}=-939,000+50\;T$	This work
Ni_2_AlLa	(Ni)_2_(Al)(La)	${\;}^{0}G_{\mathrm{Al}:\mathrm{Ni}:\mathrm{La}}^{{\mathrm{Ni}}_{2}\mathrm{AlLa}}$=$\;-205,500+0.5\;T+G_{\mathrm{Al}}^{\mathrm{fcc}}+{\;}^{0}G_{\mathrm{La}}^{\mathrm{dhcp}}+2{\;}^{0}G_{\mathrm{Ni}}^{\mathrm{fcc}}$	This work
